# An Estimation of the Potential Utilization in Iranian Pharmaceutical Industry Involved in the Stock Exchange, 2008-2012

**Published:** 2017

**Authors:** Majid Annabi, Abbas Kebriaeezadeh, Timor Mohammadi, Seyed Nasrolah Marashi Shoshtari, Farid Abedin Dorkoosh, Abolghasem Pourreza, Hassan Heydari

**Affiliations:** a *Department of Pharmacoeconomics and Pharmaceutical administration, Faculty of Pharmacy, Tehran University of Medical Sciences, Tehran, Iran. *; b *Head of the Center of Economic Research and Drug Management, Faculty of Pharmacy, Tehran University of Medical Sciences, Tehran, Iran.*; c *Pharmaceutical Management and Economics Research Center, Tehran University of Medical Sciences, Iran.*; d *Department of Theoretical Economics, Faculty of Economics, Allameh Tabataba´i University, Tehran, Iran.*; e *Department of Industrial Engineering and Management System, Faculty of Industrial Engineering, Amirkabir University of Technology,Tehran,Iran.*; f *Department of Pharmacoeconomics and Pharmaceutical Administration, Faculty of Pharmacy, Tehran University of Medical Sciences, Tehran, Iran.*; g *Department of Health Management and Economics, School of Public Health, Tehran University of Medical Sciences, Tehran, Iran.*; h *Department of Economics, Faculty of Economics and Management, Tarbiat Modares University, Tehran, Iran.*

**Keywords:** Utilized capacity, Economic potential, Pharmaceutical companies, Productivity, Stock exchange

## Abstract

The aim of this study was to measure the potential of production and the capacity used in the pharmaceutical industry. Capacity use is the actual production rate to the potential output, which reflects the gap between actual production and production capacity**.**

Through econometric methods, translog cost function in the short run along with functions of share cost of production factors is estimated through seemingly unrelated repeated regression (SURE) as a multivariate regression analysis provided by zeller.

During the study the capacity used is decreasing. The capacity used, which calculated by weighted average, also decreased and the amount during the study period is much less than the simple average of the industry. Average capacity utilization in the industry over five years of study is equal to 57% while the average capacity used calculated by the weighted of industry average is 37%. To enhance the economic potential requires a proper use of resources, creation of favorable economic structure and productivity of the industry. Due to the large amount of unused capacity in the pharmaceutical industry there is no need to invest anymore unless in new grounds and it is obvious that more investment will change using capacity.

## Introduction

Pharmaceutical companies play an important role in the health. The need for quality and easy access to drugs in the society results in the government strict supervision on the industry and products’ prices. On the other hand an important factor in determining the cost of production of goods, particularly pharmaceuticals production, is the utilization capacity which means that if the rate of exploitation is lower than utilization capacity because of the costs imposed the cost of production increase per unit, which on the other hand will reduce the firm´s profits and threaten the survival of the industry.

Although four decades have passed since the development of the pharmaceutical industry in Iran and many investments have been made ​in this sector, unfortunately so far there has been no significant action to utilize all the existing capacity in the industry efficiently (4). The difference between pharmaceutical companies in terms of productivity and its rate of change can be used as a standard basis for their economic performance. In fact, in every company, managers must not put their major reliance on working more, but rather on the efficient use of resources and higher capacities (6). Capacity is the capability of a worker, machine, work center, process, plant, or organization to produce output per period. The notion of plant capacity, for example, means the maximum amount that can be produced per unit of time with existing plant and equipment, provided that the availability of variable factors of production is not restricted )^20^, ^22^).

Due to the steady increase in production an increase in the quantity of production factors due to limitations and rareness in this case is impossible, to increase production we need to guarantee the optimum utilization of scarce resources and ensure the production function moves up which is reflected in the productivity and optimal use of all available factors (3, 5).

Capacity determination and utilization are the primary stages of capacity planning/management, which takes an important part in the area of production management (23). There are many methods used to measure the utilized capacity, one of which is the engineering method, which is very common in the industry. According to this method, installation capacity, or company establishment capacity is considered as the potential output. The method cause is problematic because it requires the full list of industrial units and updated information of the established capacity; providing these features will cost much (8). Therefore, this study was aimed to identify and measure the capacity of pharmaceutical industry using economic methods. According to the economic approach, the potential output is defined as the optimum level of production in which the average total cost curve reaches its minimum in the short run. Therefore, using this method for calculating capacity can determine the economic behavior of the pharmaceutical industry towards important input variables and can identify the cost structure and the cost share of each of the variables and their impact on the optimal production level; it also helps the pharmaceutical companies to identify strengths and weaknesses in the productivity cycle and makes it possible to plan for improving the economic status of variables, and ultimately provides the potential for the optimum use of available resources (9).

This paper is organized in several parts. First, it examines the need for measuring utilized capacity and its effect on the firm´s profits, social welfare, production costs, and consumer prices. The next part discusses the concept of utilized capacity and then presents the methodologies and the model in the study. Then it discussed the data and the selected variables, and finally the results, discussion, and conclusions.

*The concept of capacity utilized*

The concept of capacity plays an important role in the economic analysis. Unlike many of the concepts that are well defined, there are various and vague definitions of capacity. According to the Oxford business dictionary, capacity is the maximum output which can be achieved at time unit. In general, the utilized capacity is obtained from the ratio of actual output on potential output (9). Capacity utilization is usually defined as the ratio of actual output to some measure of potential output (6). In standard micro economic theory, the capacity output of a firm has been defined in several different ways. The simplest of them is the maximum level of output that can be produced from a given level of quasi-fixed inputs (like plant and machinery) even when variable inputs (like labor or materials) are available without restriction (10). For many years measurement of capacity utilized had been used to analyze the state of the economy and the effects of expansion and contraction policies. Previous studies in this area include the works by Klein, Hickman, Berndt and Morrison and Foss. These studies used the concept of capacity utilized as the short run cost function of a corporation in which one or two variables are considered to be constant (11). Many of studies on efficiency, capacity utilized are used to identify the situation and make the proper connections with the outputs (12).

Wen (25) explores the impact of capacity utilization on local determinacy in a one-sector model with a production externality. He finds that by including capacity utilization, one can obtain a locally indeterminate steady state in a one-sector model using a very mild (empirically plausible) degree of externality and at the same time have a conventional downward sloping aggregate labor demand curve) 6, 21).

Economists’ definition of the potential output is different from that expressed by the engineering perspective. From the engineering perspective, potential output can be either the establishment capacity at the beginning or the maximum capacity obtained in the previous years. An economic definition of capacity is «a level of production in which the graph of the average production reaches its minimum level». Klein provides another definition as «the highest attainable level of output of an industry in the short run without any limitation in demand using the available capital». The economic production capacity is a level of production which is consistent with the economic aspects of production, in other words, with the optimum output. The first attempt to use mathematical concepts for the examination of capacity was made by Cassel. He considered the firm’s production capacity as a condition in which the long-run average cost is minimal. This researcher suggested that the potential output calculated should be consistent with the theories of production and costs. Taking this attitude, he introduced the output with the minimum long-term production costs as the potential output. Latter, Klein notes that according to the empirical investigation, the long-term average cost function maybe L-shaped *i.e.* return to scale in the long term maybe constant; so he proposed the short run average cost function. In both methods, capacity utilized is calculated as the ratio of the actual output to the optimal output at the minimum average cost (13). From the engineering perspective, the capacity utilized means full use of resources, while from the economic perspective, the efficiency is determined by the input prices, *i.e.* the input prices and the combination of inputs are used to imply minimum cost of production. In this way, potential output (Y*) is calculated by linear regression so that the potential output (Y*) is the dependent variable and labor force, wages, raw materials, energy, and capital are the independent variables (14).

Capacity utilized is a concept in the economy that refers to the degree to which a company actually uses the installed potential output. The ratio of the actual output to the potential output capacity can show the gap in the actual output and potential output. Thus, capacity utilized is equal to the ratio of the actual output to the potential output:

Capacity utilization (CU) = Y / Y*

Where Y is the actual output and Y* is the production potential (optimum capacity or the potential output). From an engineering perspective, potential output is the maximum amount of output that could be produced in a unit of time with the existing facilities and equipments, provided that there would be no restrictions on the access to variable factors of production. From an economic point of view, potential output is the economic approach; on the other hand, the optimal output is a desired level in which, in the short run, average total cost curve reaches its minimum value (15).

*Methodology*

In terms of purpose this survey is applied research and in terms of methodology it is a descriptive research, statistical population consists of all pharmaceutical companies listed on the stock exchange the number of which is 21. Analysis of the data needed for research purposes obtained from financial companies bills listed on the stock exchange including the balance sheet and profit and loss time series in time series panel to the years between 2008-2012 as well as data of the central bank´s annual macroeconomic statistics. The data collected and processed initially into Excel software that aims to create a comprehensive database of article in order to use in other software´s. After creating a data bank at a later stage regression equation is estimated using Eviews software. Final equation is used and Matlab software calculated the potential capacity.

To estimate the optimal capacity Y* of functions and capacity utilization, short run cost function (translog) were estimated together with three cost share functions through Seemingly Unrelated Regression Estimates. In a number of experimental studies, which had been previously carried out in this field, functions were simultaneously estimated through econometric techniques. This method is a multivariate regression attributed to Zellner which is used for estimating the parameters of a system (18). This method considers variance heterogeneity and simultaneous correlation of lines for each of the equations. In other words, via weighting the residuals, this method eliminates the variance heterogeneity in cross sectional data. Since the total cost share of variable inputs is equal to a unit, and one of the equations is a linear combination of the other equations, it is not possible to estimate the model. To solve this problem, the normalization technique was used in the estimation process. Of the total 27 parameters six parameters were removed via normalization process, and it became possible to make a reverse calculation of the data matrix to estimate the parameters. Accordingly, the total variable costs and the price of the input variables are divided by on one of them (energy) and the equation will be removed. After normalization, there is no need to make restriction on the parameters (8) to make the model operational.

*Model design*

In this study, since capacity utilization was investigated using mathematical cost functions, there was a need for a reasonable and efficient model. The selected model was an experimental model, which was adopted and implemented by Berndt and Morrison in 1986 and by Nelson in 1989 (16, 17). Based on the logical premise, the logical assumption is that the connection between inputs and outputs is observed within the production function. For a company, a well-behaved function model is defined as follows:

Y = f (L, M, E, K, T)

Where Y is the output, and K, E, M, and L are capital, energy, raw materials and labor respectively and T is the time trend to distinguish technology changes in the function. From among the inputs, capital K is the constant input, and the rest are considered as variable inputs. Considering the capital as a constant input means to define short periods of time for study functions. Optimization entails maximizing profits (profit = revenue - costs); this is applied in view of output prices, input prices, and fixed capital. Following the duality theory, the optimization problem can be rewritten via minimizing variable costs (Berndt and Morrison). This is applied considering the value output of Y, prices of inputs P, capital stock K, and time trend T:

VC = f (Y, Pi, K, T)

Where VC is the total variable cost and Pi is the vector of variable input costs. To estimate the optimal output using the potential output through the above function, it is required to have a clear definition of the function. In the experimental works, the translog function is an appropriate function. In this function, the variable costs include the institutional costs of labor, raw materials, and energy. This flexible function not only covers the direct effects of the production inputs on the logarithm of the costs, but also examines the cross-logarithmic effects and square values of the variables. In this function, the substitution elasticity of factors will change in line with the changes in the factors proportions. The major advantage of this function is the flexibility of the desired parameters (2, 21). 

$\ln\mathrm{VC}=\alpha_{0}+\sum_{i=1}^{n}\ln P_{i}+0.5\sum_{i=1}^{n}i\sum_{j=1}^{n}\alpha_{\mathrm{ij}}\ln P_{i}\ln P_{j}$

$+\beta_{y}\mathrm{lnY}+0.5\beta_{\mathrm{yy}}{\left(\mathrm{lnY}\right)}^{2}+\sum_{i=1}^{n}\beta_{\mathrm{yi}}\ln Y\ln P_{i}$

$\gamma_{k}\mathrm{lnK}+0.5\gamma_{\mathrm{kk}}{\left(\mathrm{lnK}\right)}^{2}+\sum_{i=1}^{n}\gamma_{\mathrm{ki}}\ln K\ln P_{i}$

$\gamma_{\mathrm{k\gamma}}\mathrm{lnK}\ln Y_{4}+\delta_{i}T+0.5\delta_{\mathrm{ii}}T^{2}+\sum_{i=1}^{n}\delta_{\mathrm{ii}}\mathrm{Tln}P_{i}$

$+\delta_{\mathrm{tk}}\mathrm{TinK}+\delta_{\mathrm{ty}}\mathrm{TlnT}$

Usually, economic theory indicates the first order homogeneity of the cost function in the prices of production factors and odds ratios. Therefore a series of conditions should be considered on parameters.

$(a)\sum \alpha_{i}=1,\left(b\right)\sum \alpha_{\mathrm{ij}}=\sum \alpha_{\mathrm{ji}}=0$,

$\left(c\right)\sum \beta_{\mathrm{Yi}}=0\left(d\right)\sum \gamma_{\mathrm{ki}}$

Since the cost share of variables was not the same, to make a more accurate estimation, the cost share of each variable should be calculated. The cost of variable share functions can be obtained from the derivative of translog function in proportion to variable input prices with a given level of capital stock and output value. Cost ratio of each production factor is defined based on the total cost of all cost share factors.

$\frac{\partial \ln\mathrm{VC}}{\partial \ln P_{i}}=\alpha_{i}+\sum_{j=1}^{n}\alpha_{\mathrm{ij}}\ln P_{j}+\beta_{\mathrm{yi}}\mathrm{lnY}+\gamma_{\mathrm{ki}}\mathrm{lnK}+\delta_{\mathrm{ti}}T=u_{\mathrm{ji}}i=1\ldots n$

Where yi is the cost of (i) th variable. Cost share functions for every input variable together with the average short run cost are estimated to assess the cost behavior and to obtain the optimal capacity. The total short run cost includes the total variable cost and the average fixed cost. Total fixed cost is considered as the costs spent on the fixed inputs of capital. Therefore, short run total cost (SRTC) is equal to:

SRTC = VC + PKK

Short run average total cost can be obtained via dividing the above equation by the output value:

SATC = (VC / Y) + (PKK / Y)

Finally, the optimal capacity (Y = Y*) is obtained where SATC is minimal, *i.e.* where the derivative of the function is zero, then (∂ SATC/∂ Y) = 0. Given the above equation, we have:

1Y*∂VC∂Y*-VCY*2-PKKY*2=0(1)

Where ∂VC∂Y*=∂lnVC∂lnY*VCY*

and

∂lnVC∂lnY*=βy+βyylnY*+∑i=1nβyilnPi+γkylnK+δtyT=μy (2)

Substituting (1) in (2),

$\frac{\partial \mathrm{sATC}}{\partial Y^{*}}=\mathrm{VC}\left(\mu_{y}-1\right)-P_{k}K=0$

Since yi and VC are both a function of Ln Y* and Y*, the optimal output value cannot be estimated from the closed model. To resolve this problem, approximate equations can be used for obtaining optimum output (15).

**Table 1:** Descriptive statistics of the data

**Min**	**Max**	**Standard** **deviation**	**Average**	
2,979	82,877	16,646	22,357	Capital Stock
11,931	145,540	24,687	37,568	Labor cost
0.01	0.19	0.04	0.09	Capital cost
265	4,955	929	1,285	total cost of fuel
79	295	44	164	wage rate
14,773	450,148	104,372	164,319	total cost of material
89,428	1,006,246	215,995	355,051	Output
45,755	519,948	119,353	203,172	Total variable cost
47,910	522,491	120,013	204,782	Total cost

**Table 2:** Trends of variable changes in the pharmaceutical industry of Iran (2008-2012

**2012**	**2011**	**2010**	**2009**	**2008**	
20,202	22,991	26,107	22,916	19,570	Capital Stock
32,746	40,186	41,647	38,681	34,581	Labor cost
0.09	0.09	0.08	0.09	0.09	Capital cost
1,636	1,793	1,060	939	997	total cost of fuel
212	183	161	144	122	wage rate
126,985	171,113	170,235	187,825	165,436	total cost of material
273,579	368,604	383,594	414,302	335,177	Output
161,367	213,092	212,942	227,444	201,014	Total variable cost
162,829	214,728	214,586	229,139	202,627	Total cost

**Table 3.** Seemingly unrelated regression estimates for translog cost function

**parameter**	**Coefficient**	**t-Statistic**	**parameter**	**Coefficient**	**t-Statistic**	
$\alpha_{0}$	11.62775	1.565971	$\gamma_{\mathrm{kk}}$	0.016730	0.305646	
$\alpha_{k}$	1.406375	1.533723	$\gamma_{\mathrm{kk}}$	-0.009104	-0.238697	
$\alpha_{l}$	-0.970552	-0.706229	$\gamma_{\mathrm{kl}}$	-0.063675	-0.977814	
$\alpha_{m}$	0.876535	1.001358	$\gamma_{\mathrm{km}}$	0.041262	0.623188	
$\alpha_{\mathrm{kl}}$	-0.275895	-2.136085	$\gamma_{\mathrm{k\gamma}}$	-0.007069	-0.136643	
$\alpha_{\mathrm{km}}$	0.105622	0.736631	$\delta_{T}$	0.826422	2.828741	
$\alpha_{\mathrm{lm}}$	0.305991	1.367267	$\delta_{\mathrm{TT}}$	0.025478	1.300421	
$\beta_{y}$	0.388660	0.360616	$\delta_{\mathrm{Tk}}$	-0.004992	-0.216582	
$\beta_{\mathrm{yy}}$	-0.011962	-0.144897	$\delta_{\mathrm{Tl}}$	0.040607	0.807601	
$\beta_{\mathrm{yk}}$	-0.077406	-1.097104	$\delta_{\mathrm{Tm}}$	0.085243	2.705856	
$\beta_{\mathrm{yl}}$	0.082493	0.772533	$\delta_{\mathrm{tk}}$	0.003814	0.200494	
$\beta_{\mathrm{ym}}$	-0.039079	-0.596516	$\delta_{\mathrm{ty}}$	-0.027046	-1.168214	
$\gamma_{k}$	0.130004	0.186804	DW	1.098214		

Note: DW indicates Durbin-Watson Statistic.

**Table 4:** The potential output and actual output in the pharmaceutical industry in Iran (2008-2012).

**2012**	**2011**	**2010**	**2009**	**2008**	**Year**
16,224,200	22,213,200	19,127,800	25,798,800	15,068,660	Potential Output (Y*)
5,471,573	7,372,084	7,671,876	8,286,043	6,703,543	Actual Output (Y)

**Table 5:** Average capacity utilization and the weighted average capacity utilization in the pharmaceutical industry (2008-2012

**Average**	**2012**	**2011**	**2010**	**2009**	**2008**	**year**
57%	52	46	61	61	66	Capacity utilization (Average of industry)
37%	34	33	40	32	44	Capacity utilization (Weighted average of industry)

*Data and variables*

Data used in this study included the time series panel data, which was collected from pharmaceutical companies in the stock exchange, between the years 2008–2012. The data was taken from the official financial statements and audit information of the companies. Tables 1 and 2 respectively show the descriptive statistics of model variables and their changes over the years of the study.

Output: Gross output value which was deflated by the producer price index (PPI). Gross output value was selected to eliminate the differences in the calculation methods, and assumptions were separately accredited to use value-added.

Capital stock: Capital stock had become factual by the deflator implicit indicator which is achieved through dividing the asset capital of the year by the fixed capital of 1997.

Capital cost: it is a cost which was spent on improvements and fundamental repairs in tangible fixed assets to substantially increase the capacity, the operational life, the quality, or their efficiencies. Varying cost of the entire industry: it included the total cost of labor, raw materials, energy, and amortization expense (24).

Labor cost and wage rate: it was the value of all payments to all employees in the pharmaceutical industry, including all personnel working in manufacturing and staff units. Per capita cost of labor was achieved via dividing compensation cost of labor by the number of personnel. Labor cost was deflated by the wage increase index.

Cost Energy*:* it was deflated by the price increase index of all components of the energy value and in proportion with the share of each of these components in the total energy costs. Energy costs included industry expenses on electricity, oil, natural gas, petrol, and diesel. To calculate the energy price index, the weighted average price rise of energy was considered for all components of energy costs.

Total cost of material*:* it was the value of all the materials including pharmaceutical raw materials and packaging supplies used in manufacturing drugs which was deflated by the producer price index (PPI). 

## Results

After entering the data into Excel software and processing them, they were transferred to Eviews software and gradually we estimated the practical model of cost function and cost share functions simultaneously. The basic translog cost function had 27 parameters, and after normalization six parameters were removed and 21 parameters remained. Table 3 presents the results of estimating the short run cost function parameters. Coefficient of determination or R2 is a good measure for comparing the validity of the results of logistic regression models. The obtained values from estimation models for the variable cost function equation, cost share equation, labor cost equation, raw material cost share equation, and the capital cost equation were 0.96, 0.89, 0.82, and 0.84, respectively, which shows a very good explanation for the estimated model. In other words, the explanatory variables show 96% of changes in the dependent variable. To determine the status of the return to scale, the parametric restrictions imposed to the scale were examined using chi-square test. However, a cost function is well behaved only if it is concave in input prices and if its input shares function is positive. Generally, the first statistic test, which is commonly used to evaluate the significance of regression coefficients, is *t*-test. This test is a method that uses a sample of the results, to determine whether the null hypothesis is right or wrong. This statistic either accepts or rejects the impacts of each exploratory variable on the dependent variable. According to the table, most of the calculated coefficients are against zero, with a 95% significance level. The high number of significant coefficients in the model without very large and very small values for the parameters indicates that there is no co-linearity between the explanatory variables. Coefficients of the translog cost function are not significant by themselves; however they can help to determine the optimum output. To use this model, the parameters of the model should be consistent with economic theories. The positive sign of cost function coefficients shows ascending trend in proportion to the production levels and the input prices. According to the estimated model, estimated parameters are not only statistically significant but are also economically acceptable.

Table 4 shows the amount of potential output and actual output in the pharmaceutical companies during the years of the study. There is no regularly growing trend in the potential output. The maximum amount of optimum output was observed in 2009. The actual output of companies had a decreasing trend during the study period and its maximum level was observed in 2009.

The ratio of the actual output to the potential output shows the gap between the actual output and the potential output. So, it is equal to the relationship between the actual output to the potential output (CU = Y / Y*). Table 5 shows the capacity utilization in the pharmaceutical industry in two forms of the industry average and the weighted average. Capacity utilization had been declining during the study years. Despite the mass production, Return on sales, which is the ratio of net profit to sales, was reduced because of the decline in economic capacity. Capacity utilization which was calculated by the industry weighted average had also a decreasing trend and its value had been always much less the industry weighted average over the years of the study. Average capacity utilization during the five years of the study was 57%, while the average capacity utilization calculated by the industry weighted average was 37% in the same period (Table 5).

## Discussion

The ratio of Capacity utilization provides useful information for firms which have low profit margin products; it also provides the opportunity for an investment decision. Low capacity utilization in the pharmaceutical industry indicates that there is no need for further investments and any additional investment only increases the financial burdens on the industry (4). Reduced capacity utilization indicates a lack of logical optimization processes and proves that the productivity system does not exist in the pharmaceutical companies. Low capacity utilization and its decreasing trend can increase the unused capacity and thereby increase the financial burden of companies, especially large companies.

Since the economic structure of a company has an impact on the economic capacity and the capacity utilization is calculated within a framework of economic optimization, it represents the current economic structure and environmental variables and provides useful information (18). Low capacity utilization in the companies in the Iranian pharmaceutical industry implies that the companies do not have the right cost structure and the costs are not managed at all; additionally, despite mass production, return on sales, which is the ratio of net income to sales, has decreased due to the decline in the economic capacity. The significant difference between average capacity utilization with the weighted average capacity utilization shows that the major pharmaceutical companies in Iran have lower capacity utilization than small companies; large companies must have mechanisms to control costs in order to be able to use all available capacity, so that they earn profits commensurate with the amount of used assets. Proper management of human resources and raw materials and the correct use of capital can help companies increase their economic capacity.

The results of the estimated coefficients of the cost function parameters prove the fact that the behavior of pharmaceutical companies isn’t an economic behavior and the trends and the impact of input prices are not logically related to the costs, production rates, capital, and technology; such a phenomenon might be perhaps due to some factors such as hiding the actual costs of the company and inefficiency of the management in these companies. On the other hand, cost share equation which represents the firm´s behavior toward used resources and their mutual impact on each other, and the estimated coefficients of the cost function parameters all prove the fact that economic behavior of the pharmaceutical companies is an economic behavior in this regard.

The effects of input price fluctuations on the potential output and capacity utilization depend on the substitution of the inputs with the capital (18). In this study, there was a positive relationship between capacity utilization and the inputs (labor, raw materials, and capital), *i.e.* with a decrease in the prices of raw materials, the capacity utilization was likely to increase; as a result of the economic conditions of a society could affect the capacity utilization. As observed in Table 5, in 2011 and 2012 the economic condition was under the influence of currency fluctuations, hence it can be an important factor in decreeing the capacity utilization. In addition to the prices of inputs, output can also affect the capacity utilization. In market equilibrium, increased output can be considered as a demand factor; in other words, an increase in the demand can lead to increased capacity utilization (20).

González and Gascón (26), in an article titled, “Sources of productivity growth in the Spanish pharmaceutical industry (1994–2000)”, estimate Malmquist productivity indexes and decompose them into four sources of productivity change. The results suggest that absolute technical efficiency change and the scale change of the technology explain most of the productivity growth observed during the period. The results of this paper are consistent with this paper. Also Nghiem and Coelli (27) in their paper decompose productivity growth into useful components, including technical efficiency changes, technological changes and scale changes. The results revealed an average of 1.6 per cent of growth in total factor productivity. And component contributing to the modest improvement of TFP during the period was catching-up at an average of 1.0 per cent. This study is not in line with the results of this paper. In a study by Azeez (28), he attempts to estimate a stable series for the economic capacity utilization of the Indian non-electrical machinery manufacturing sector. Capacity utilization (CU), defined as the ratio of actual to optimal output, is the systematic results of logical optimization procedures, depending on price and cost conditions of firms. A comparison of new series with the conventional engineering measures of CU shows that the widely used «installed CU” figures clearly underestimate the correct economic utilization levels, mainly because of definitional problems.

## Conclusion

In this study, we examined the potential output and capacity utilization of the pharmaceutical companies present in the stock market from 2008 to 2012. We used translog short run cost function to estimate the output, so that short run cost curve would be minimal. The rate of capacity utilization was low and had a decreasing trend over the years of study. To enhance the economic capacity, it is required to use resources efficiency or to have a productive system in the industry. Translog equation expresses the mutual relationships between costs, revenues, and the profitability of an industry. The results of the estimation of the parameters and coefficients represent the fact that the behavior of pharmaceutical companies is not an economic behavior and the costs are not shown realistically. The behavior of the companies can be better studied via classifying companies, for instance into public companies or private companies or they can be classified based on their size. On the other hand, cost share equation which represents the firm´s behavior toward used resources and their mutual influence on each other and the results of the estimated coefficients of the cost function parameters prove the fact that the behavior of pharmaceutical companies is an economic behavior. Given the data and the equations which can be used to calculate cost elasticity, the cost structure of selected companies can be analyzed in further researches. Given the large untouched capacity in the pharmaceutical industry, there is no need to invest except for the new and innovative fields, where further investments alter the capacity utilization. The lack of reliable data in all companies has led us to include only those companies present in the stock exchange market whose data are investigated by the independent audit companies. Also, previous research and empirical evidence related to the issue in most cases show, Estimates used in the pharmaceutical industry´s capacity is low and has been decreasing during the years of study that. 
